# Supplementary material for: CA9, CYFIP2 and LGALS3BP—A Novel Biomarker Panel to Aid Prognostication in Glioma
Source: Cancers (Basel). 2024 Mar 6;16(5):1069. doi: 10.3390/cancers16051069 (PMC10931055; doi:10.3390/cancers16051069)
Supplement: Supplementary file 1 [file cancers-16-01069-s001.zip › cancers-2848667-supplementary.pdf]

SUPPLEMENTARY MATERIAL

Title: *CA9, CYFIP2 and LGALS3BP - a novel biomarker panel to aid prognostication in glioma*

Authors: Amanda L. Hudson [1,2,3*†], Angela Cho [1,2,3†], Emily K. Colvin [1,2], Sarah A. Hayes [1,2], Helen R. Wheeler [3,4] and Viive M. Howell [1,2].

[1] Bill Walsh Translational Cancer Research Laboratory, Kolling Institute, Royal North Shore Hospital, Northern Sydney Local Health District, St. Leonards, 2065, Australia
[2] School of Medical Sciences, Faculty of Medicine and Health, University of Sydney, Sydney, NSW, 2006, Australia
[3] The Brain Cancer Group, North Shore Private Hospital, St. Leonards, NSW, 2065, Australia
[4] Department of Medical Oncology, Royal North Shore Hospital, Northern Sydney Local Health District, St. Leonards, NSW 2065, Australia

Supplementary Figures and Table list

Figure S1: Representative images of the 6 candidate biomarkers in glioma tissue sections and their different scoring results.

Figure S2: Interaction schematic of the CA9, CYFIP2 and LGALS3BP using STRING.

Table S1: Clinical characteristics of discovery cohort.

Table S2: Immunohistochemistry antibodies.

Table S3: Cut off points for defining low and high protein expression by IHC.

Table S4: Differentially expressed proteins identified in discovery cohort.

Table S5: Cancer related functions and association with prognosis of candidate biomarkers.

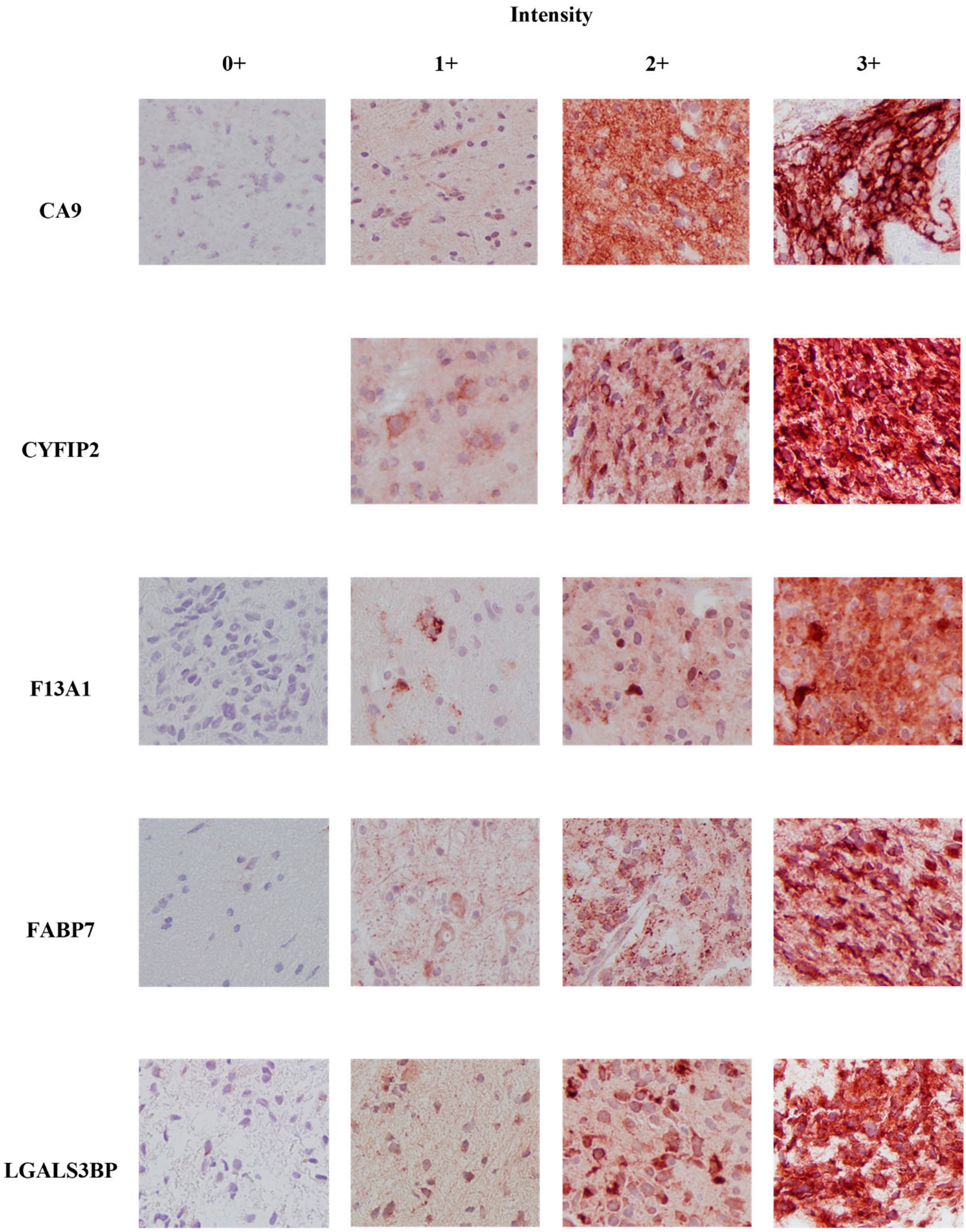

Figure S1: Representative images of the 6 candidate biomarkers in glioma tissue sections and their different scoring results. 20 × magnification.

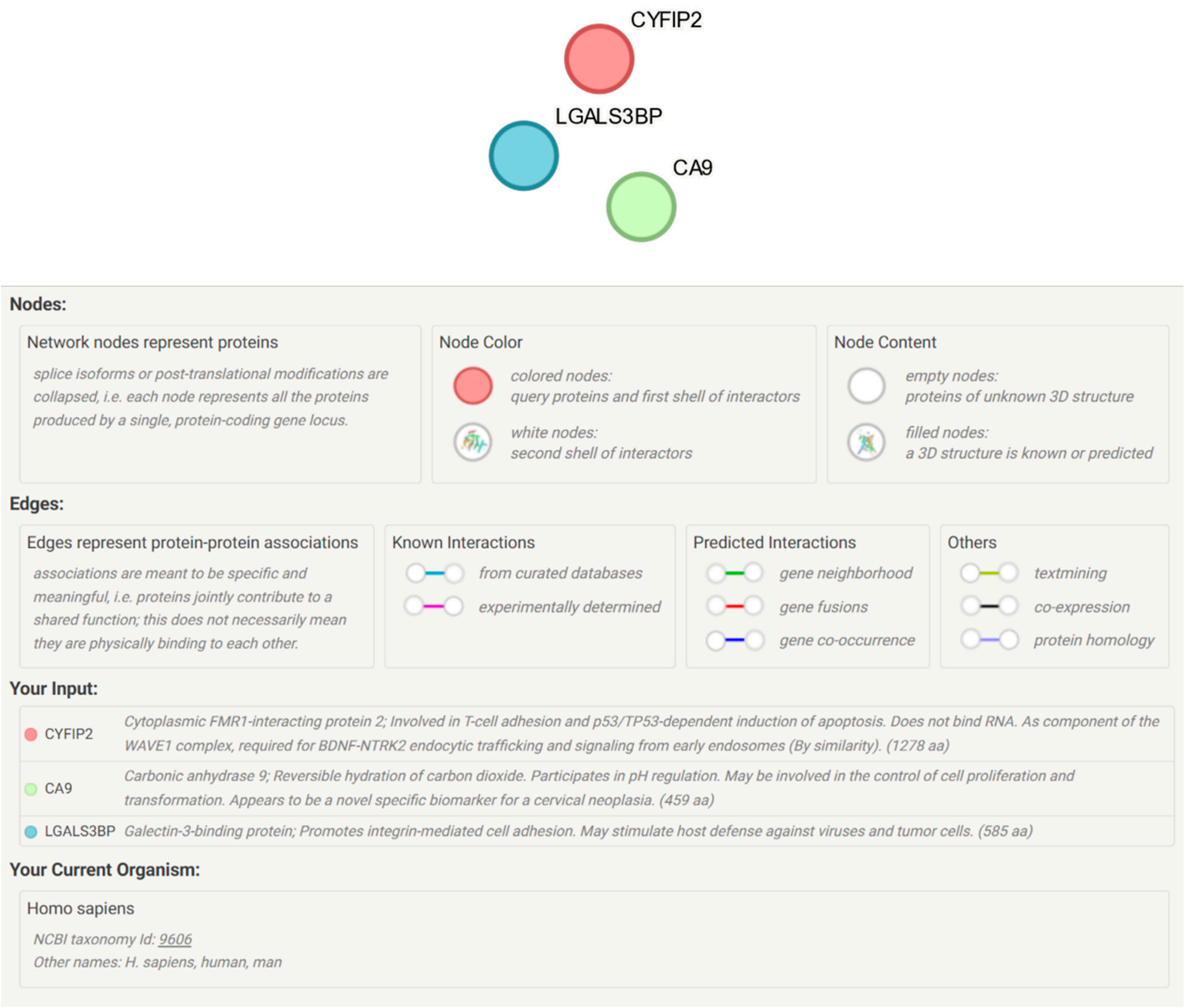

SI Figure 2: Interaction schematic of the CA9, CYFIP2 and LGALS3BP using STRING (accessed 5th December 2023) confirming no published interaction have been identified [1].

Table S1: Clinical characteristics of discovery cohort.

	**Number of patients (%)**
**Gender**	
**Female**	4 (66.7)
**Male**	2 (33.3)
**Age at diagnosis (years)**	
**Median**	38
**Range**	30-46
IDH	
IDH1 R132H	5 (83.3)
IDH1 R132G	1 (16.7)
**Primary tumour grade**	
**II**	4 (66.6)
**III**	2 (33.3)
**Recurrent tumour grade**	
**III**	1 (16.6)
**IV**	5 (83.3)
**Treatment (in between specimens)**	
**Radiotherapy**	3 (50)
**None**	3 (50)
**Time to recurrence (Months)***	
**Median**	46
**Range**	14-99
**Outcome#**	
**Alive**	4 (66.7)
**Dead**	2 (33.3)

*calculated as the months between the primary and recurrent surgery; #censor date, 29th Feb 2020.

Table S2: Immunohistochemistry antibodies.

Specificity	Host species/Isotype	Concentration (µg/ml)	Company	Catalogue number
**CA9 (carbonic anhydrase 9)**	Rabbit IgG	0.5	Abcam	Ab15086
**CYFIP2 (cytoplasmic FMR1-interacting protein 2)**	Rabbit IgG	2.5	Life Technologies	PA568449
**F13A1 (Coagulation factor XIII A chain)**	Rabbit IgG	0.25	Sigma-Aldrich	HPA001804
**FABP7 (Fatty acid-binding protein, brain)**	Rabbit IgG	0.25	Sigma-Aldrich	HPA028825
**LGALS3BP (galectin-3-binding Protein)**	Rabbit IgG	0.25	Sigma-Aldrich	HPA000554
**PRKAR1A (cAMP-dependent protein kinase type I-alpha regulatory subunit)**	Rabbit IgG	1	Sigma-Aldrich	HPA049979

Table S3: Cut off points for defining low and high protein expression by IHC.

Protein (Cellular localisation)	Expression level	Concordance score (Tumour cells staining (%) × intensity)
**CA9 (membranous/ cytoplasmic)** **F13A1 (cytoplasmic)**	None	0
	Any (Low- high)	> 0
**CYFIP2 (cytoplasmic)** **FABP7 (cytoplasmic)**	Low	≤ 200
	High	>200
**LGALS3BP (cytoplasmic)**	Low	≤ 95
	High	>95
**PRKAR1A (cytoplasmic)**	Low	≤ 40
	High	> 40

Table S4: Differentially expressed proteins identified in discovery cohort. *Samples were further stratified into patients that received no treatment (A) and those that received radiotherapy (B) in between their surgeries. P-value <0.05 indicates significant results.*

Direction of change*	Comparison	Uniprot ID	Protein Name	Protein abbreviation	Log 2 FC	P - value
UP	A	Q16527	Cysteine and glycine-rich protein 2	CSRP2	2.09	0.027
	A	Q08380	Galectin-3-binding protein	LGALS3BP	1.52	0.024
	A	P04004	Vitronectin	VTN	1.43	0.034
	A	Q6IAA8	Ragulator complex protein	LAMTOR1	1.10	0.035
	A	P01042	Kininogen-1	KNG1	1.02	0.016
	B	P00488	Coagulation factor XIII A chain	F13A1	1.39	0.044
DOWN	A	Q9NZJ7	Mitochondrial carrier homolog 1	MTCH1	-1.07	0.013
	A	O00429	Dynamin-1-like protein	DNM1L	-1.08	0.032
	A	P31946	14-3-3 protein beta/alpha	YWHAB	-1.15	0.012
	A	P08195	4F2 cell-surface antigen heavy chain	SLC3A2	-1.19	0.013
	A	P07954	Fumarate hydratase	FH	1.21	0.014
	A	P21796	Voltage-dependent anion-selective channel protein 1	VDAC1	-1.23	0.007
	A	Q9P2R7	Succinate-CoA ligase	SUCLA2	-1.24	0.015
	A	Q9H936	Mitochondrial glutamate carrier 1	SLC25A22	-1.28	0.040
	A	O43301	Heat shock 70 kDa protein 12A	HSPA12A	-1.31	0.045
	A	Q99798	Aconitate hydratase	ACO2	-1.31	0.033
	A	O75306	NADH dehydrogenase	NDUFS2	-1.36	0.005
	A	O15540	Fatty acid-binding protein	FABP7	-1.38	0.008

	A	Q96F07	Cytoplasmic FMR1-interacting protein 2	CYFIP2	- 1.43	0.024
	A	P46459	Vesicle-fusing ATPase	NSF	- 1.65	0.045
	A	P68366	Tubulin alpha-4A chain	TUBA4A	- 1.69	0.046
	A	Q9BRX8	Redox-regulatory protein FAM213A	PRXL2A	- 1.81	0.018
	A	Q99719	Septin-5	SEPTIN5	- 1.85	0.034
	A	P80723	Brain acid soluble protein 1	BASP1	- 1.87	0.048
	B	P13637	Sodium/potassium-transporting ATPase subunit alpha-3	ATP1A3	- 1.02	0.004
	B	P62873	Guanine nucleotide-binding protein	GNB1	- 1.06	0.044
	B	P37840	Alpha-synuclein	SNCA	- 1.07	0.006
	B	P10644	cAMP-dependent protein kinase type I-alpha regulatory subunit	PRKAR1A	- 1.09	0.013
	B	P60880	Synaptosomal-associated protein 25	SNAP25	- 1.13	0.011
	B	P08247	Synaptophysin	SYP	- 1.14	0.007
	B	P17600	Synapsin-1	SYN1	- 1.41	0.002
	B	P63027	Vesicle-associated membrane protein 2	VAMP2	- 1.50	0.006

**in recurrent samples.*

Table S5: Cancer related functions and association with prognosis of candidate biomarkers.

Protein name (Symbol, Uniprot ID)	Function	Associations with cancer	References
**Carbonic anhydrase 9 (CA9, Q16790)**	pH regulation; hypoxia inducible	Glioma - silencing of *CA9* reduced invasion, enhanced susceptibility to temozolomide (TMZ) and radiotherapy *in vitro;* inhibition of CA9 enhances pro-apoptotic effects of TMZ; overexpression is associated with poorer overall survival.	[12, 13, 28, 43]
**Cytoplasmic FMR1-interacting protein 2 (CYFIP2, Q96F07)**	Pro-apoptotic protein and a direct target of TP53. Involved in T-cell adhesion.	Repression of CYFIP2 promoted colon cancer cell proliferation, growth and survival. Knockdown promoted proliferation, chemoresistance and inhibited apoptosis in gastric cancer cells.	[34, 44]
**Coagulation factor XIII A chain (F13A1, P00488)**	Key component in the coagulation cascade. F13A1 cross-links fibrin chains and protects them from fibrinolysis to strengthen the fibrin clot.	High expression associated with progression in lung squamous carcinomas. Downregulated in tumour-educated platelets in non-small cell lung cancer. Plasma levels in ovarian cancer patients were significantly higher compared to controls. Serum levels of the activation peptide of F13A1 were significantly reduced in colorectal patients.	[45-49]
**Fatty acid-binding protein, brain (FABP7, O15540)**	Important in the formation of the radial glial fibre in the developing brain.	Glioma - highly expressed in grade IV gliomasU251 glioma cells Overexpression or knockdown in glioma cell line U251 promoted or reduced proliferation, respectively. In glioma neurospheres, expression increased after radiation and resulted in enhanced migration and proliferation.<br><br>Melanoma, breast cancer, renal cell carcinoma - overexpressed and associated with proliferation, higher	[50-57]

		grade of malignancy and shorter overall survival.	
**Galectin-3-binding protein (LGALS3BP, Q08380)**	Essential role in centrosome structure and function; modulates cell-cell and cell-matrix interactions.	Overexpressed in prostate, colorectal and epithelial ovarian cancers. Overexpression inhibits neutrophil-mediated tumour cell killing in colorectal cells.<br><br>Breast cancer – over expression promotes angiogenesis and metastasis; associated with poor survival.	[58-62]
**cAMP-dependent protein kinase type I-alpha regulatory subunit (PRKAR1A, P10644)**	Regulatory subunit of the cyclic adenosine monophosphate (cAMP)- dependent protein kinases involved in cAMP signaling in cells.	Potential tumour suppressor.<br><br>Lung adenocarcinoma - low expression correlated with poor overall survival.<br><br>Inactivating mutations causes Carney Complex (patients develop multiple benign tumours).	[63-67]
